# Whole genome sequencing in adults with clinical hallmarks of hypophosphatasia negative for *ALPL* variants

**DOI:** 10.1007/s11033-024-09906-7

**Published:** 2024-09-14

**Authors:** Lothar Seefried, Anna Petryk, Guillermo del Angel, Felix Reder, Peter Bauer

**Affiliations:** 1 Clinical Trial Unit, Orthopedic Department, University of Würzburg, Brettreichtstr. 11, 97074 Würzburg, Bavaria, Germany; 2 Alexion, AstraZeneca Rare Disease, Boston, MA USA; 3 Centogene GmbH, Rostock, Germany

**Keywords:** Hypophosphatasia, Rare disease, Diagnosis, Genetics, Whole genome sequencing

## Abstract

**Background:**

Hypophosphatasia (HPP) is a rare disease caused by deficient activity of tissue-nonspecific alkaline phosphatase (ALP), encoded by the *ALPL* gene. The primary objective was to explore novel *ALPL* variants by whole genome sequencing (WGS) in patients with HPP who previously tested negative by standard methods for *ALPL* variants. The secondary objective was to search for genes beyond *ALPL* that may reduce ALP activity or contribute to HPP symptoms.

**Methods and results:**

WGS was performed in 16 patients clinically diagnosed with HPP who had ALP activity below the normal range and tested negative for *ALPL* variants. Genetic variants in *ALPL* and genes possibly associated with low ALP activity or phenotypic overlap with HPP were assessed. All 16 patients had ALP activity below the normal range. WGS did not identify any novel disease-causing *ALPL* variants. Positive findings for other gene variants were identified in 4 patients: 1 patient presented with variants in *COL1A1*, *NLRP12*, and *SCN9A*, coding for collagen, type, I alpha-1 chain, nod-like receptor pyrin domain containing 12, and sodium voltage-gated channel alpha subunit 9, respectively; 1 presented with a heterozygous, likely pathogenic variant in *P3H1* coding for prolyl 3 hydroxylase 1; 1 presented with a heterozygous pathogenic variant in *SGCE*, coding for sarcoglycan epsilon; and 1 presented with a heterozygous variant of uncertain significance in *VDR*, encoding vitamin D receptor.

**Conclusion:**

Genomic analysis did not identify novel *ALPL* variants or a pattern of disease-causing variants in genes other than *ALPL* to explain the HPP phenotype in these patients.

**Registration:**

Clinicaltrials.gov identifier: NCT04925804.

## Introduction

Hypophosphatasia (HPP) is a rare, metabolic disease caused by deficient activity of the enzyme tissue-nonspecific alkaline phosphatase (ALP), encoded by the *ALPL* gene [1]. Low ALP activity may result in accumulation of its substrates, including inorganic pyrophosphate (PPi) and pyridoxal 5ʹ-phosphate (PLP). The accumulation of PPi, a potent inhibitor of bone mineralization, can cause defective skeletal mineralization, while impaired dephosphorylation of PLP into pyridoxal can result in vitamin B_6_–responsive seizures [1, 2]. While the clinical spectrum of HPP is extremely broad across different age groups, it is often associated with severe systemic morbidity, particularly due to musculoskeletal deficits (e.g., fractures, muscle weakness) and pain, in patients manifesting the disease later in life [3].

Accurate, timely diagnosis of HPP is important for appropriate disease management [1]. A clinical diagnosis of HPP is based on identification of characteristic manifestations [1]. Differential diagnosis involves the exclusion of other pathologic conditions associated with phenotypic overlap and/or low ALP activity [1]. Sequencing of the *ALPL* coding region can be used to confirm a diagnosis of HPP [1] and identifies variants in approximately 95% of patients with the disease [4]. To date, more than 400 variants of the *ALPL* gene, predominantly missense variants, have been identified [5], indicating substantial allelic heterogenicity. However, standard sequencing techniques may fail to detect *ALPL* variants in some patients because of the presence of new variants not yet classified as pathogenic, variants within introns or within the regulatory sequences of *ALPL*, large deletions/duplications not typically assessed by standard methods, or variants in other genes involved in the regulation of *ALPL* [6, 7].

The primary objective of this study was to identify novel *ALPL* variants by whole gene sequencing (WGS) in patients with HPP who previously tested negative by standard methods for *ALPL* variants. The secondary objective was to search for genes beyond *ALPL* that may reduce ALP activity or contribute to HPP symptoms in these patients.

## Materials and methods

### Objectives

The primary study objective was to compare the results of WGS with those of standard sequencing carried out for detection of *ALPL* variants. The secondary objective was to search for gene variants within or beyond *ALPL* that could result in low ALP activity.

### Patients

This prospective, observational cohort study (NCT04925804) was conducted between June 2 and December 2, 2021, at a single HPP expert center at University of Würzburg, Germany. Eligible patients had a clinical diagnosis of HPP based on age- and sex-adjusted low serum ALP activity and clinical symptoms. ALP activity was determined on 2 occasions, at least 1 month apart, using a commercially available assay. Patients were required to have an available sequencing report indicating no pathogenic variants, no likely pathogenic variants, or no variants of uncertain significance (VUS) in *ALPL*. Patients were excluded if they had other potential causes of low ALP, including celiac disease, clofibrate therapy, cleidocranial dysplasia, Cushing’s syndrome, hypothyroidism, massive blood transfusion, milk-alkali syndrome, multiple myeloma, osteogenesis imperfecta type II, pernicious or profound anemia, starvation, vitamin C deficiency, vitamin D intoxication, zinc deficiency, and magnesium deficiency.

For each patient, medical history was obtained using a standardized questionnaire indicating specified signs and symptoms as present or absent, and entering the history and date of use of specified medications. Data were collated by means of an electronic case report form. Patient-level data were de-identified by assigning project-specific codes (i.e., patient numbers) to the clinical data and biologic specimens. The protocol and an amendment were approved by the ethics committee of the medical faculty of the University of Rostock (Vote number A2020-0169) as well as by the ethics committee of the University of Würzburg (Vote number 221/20). The study was conducted in compliance with guidelines for Good Clinical Practice and the ethical principles of the Declaration of Helsinki. Written informed consent was obtained from patients prior to initiation of study procedures.

### WGS

Kits containing the Centogene GmbH (Rostock, Germany) proprietary dry blood spot card (CentoCard) were used to collect blood samples from patients and were returned to Centogene for analysis. Genomic DNA was isolated from the CentoCard using a standard protocol developed by Centogene. After fragmentation of genomic DNA by sonication, Illumina adapters were ligated to generate fragments for subsequent sequencing on the HiSeqX platform (Illumina, Inc., San Diego, CA, USA) to yield an average coverage depth of more than 30X.

Genetic testing was performed to detect *ALPL* variants that are pathogenic/likely pathogenic/VUS and rare in the general population. The *ALPL* gene (including exons, introns, and regulatory sequences) was evaluated, as well as a panel of genes (Table 1) directly or indirectly related to *ALPL*, *ALPL* regulation, or the HPP phenotype based on available evidence in the literature [1, 8, 9]. Variant calls included single nucleotide variants (SNVs; deep intronic variants with or without splice predictions were also considered), copy number variants (CNVs), and any other rearrangements (e.g., inversions, gross insertions, translocations). All potentially relevant variants detected in *ALPL* or any additionally paneled genes are reported herein, including variants found in reference population datasets (e.g., gnomAD).

**Table 1:** Candidate genes that may regulate ALP activity or modify the HPP phenotype

Diseases with Phenotypic Overlap		
Disease	Expected ALP Enzyme Activity	Gene(s)
Osteogenesis imperfecta	Normal or elevated [1]	*BMP1*, *COL1A1*, *COL1A2*, *CREB3L1*, *IFITM5*, *MBTPS2*,* SERPINH1*, *SP7*, *SPARC*, *TENT5A*, *TMEM38B*, *WNT1*
Cole-Carpenter syndrome	Normal	*P4HB*, *SEC24D*
Dentinogenesis imperfecta	Normal	*DSPP*
Vitamin D-dependent rickets	Elevated	*CYP27B1*, *VDR*
Tumoral calcinosis	Elevated	*FGF23*
Campomelic dysplasia	Normal	*SOX9*
Hadju-Cheney syndrome	Normal	*NOTCH2*
Cleidocranial dysplasia	Normal or reduced [25]	*RUNX2*
Hypophosphatemic rickets	Elevated	*PHEX*, *SLC37A3*
Stüve-Wiedemann syndrome	Normal	*LIFR*
**Modifier Genes of HPP**		
**Study population**	**Gene(s)**	
46 patients with HPP, ALP not systematically available [8]	*ANKH*, *ENPP1*, *FGFR3*,* PHOSPHO1*, *PTH1R*,* PTH2R*, *SPP1*, *TNFRSF11A*	
Population-based GWAS (various populations) [9]	*GPLD1*	
**Gene Panels**		
**Panel**	**Genes**	
Blueprint Genetics (Skeletal Dysplasia with Abnormal Mineralization panel)	*ALPL*, *ANKH*, *B4GALT7*, *CASR*, *CLCN5*, *COL1A1*, *COL1A2*, *COL3A1*, *COL5A1*, *COL5A2*, *CRTAP*, *CYP27B1*, *ENPP1*, *FBN1*, *FGF23*, *FKBP10*, *GALNT3*, *MGP*, *P3H1*, *PHEX*, *PLOD2*, *PLS3*, *PPIB*, *PTDSS1*, *SERPINF1*, *SLC34A3*, *SLC39A13*, *SNX10*, *SOX9*, *TNFRSF11A*, *TNFRSF11B*, *VDR*	
GeneDx (Abnormal Mineralization panel)	*ALPL*, *ANKH*, *AP2S1*, *CASR*, *CLCN5*, *CYP27B1*, *CYP2R1*, *DMP1*, *ENPP1*, *FAH*, *FGF23*, *PHEX*, *SLC34A1*, *SLC34A3*, *SLC9A3R1*, *VDR*	

ALP, alkaline phosphatase; GWAS, genome-wide association study; HPP, hypophosphatasia

Genes: *ALPL*, alkaline phosphatase, tissue-nonspecific; *ANKH*, ANKH inorganic pyrophosphate transport regulator; *AP2S1*, adaptor-related protein complex 2, sigma-1 subunit; *B4GALT7*, beta-1,4-galactosyltransferase 7; *BMP1*, bone morphogenetic protein 1; *CASR*, calcium-sensing receptor; *CLCN5*, chloride voltage-gated channel 5; *COL1A1*, collagen, type I, alpha-1 chain; *COL1A2*, collagen, type I, alpha-2 chain; *COL3A1*, collagen, type III, alpha-1 chain; *COL5A1*, collagen, type V, alpha-1 chain; *COL5A2*, collagen, type V, alpha-2 chain; *CREB3L1*, cAMP responsive element-binding protein 3-like 1; *CRTAP*, cartilage-associated protein; *CYP2R1*, cytochrome P450, family 2 subfamily R member 1; *CYP27B1*, cytochrome P450, family 27 subfamily B member 1; *DMP1*, dentin matrix acidic phosphoprotein 1; *DSPP*, dentin sialophosphoprotein; *ENPP1*, ectonucleotide pyrophosphatase/phosphodiesterase 1; *FAH*, fumarylacetoacetate hydrolase; *FBN1*, fibrillin 1; *FGF23*, fibroblast growth factor 23; *FGFR3*, fibroblast growth factor receptor 3; *FKBP10*, FKBP prolyl isomerase 10; *GALNT3*, polypeptide N-acetylgalactosaminyltransferase 3; *GPLD1*, glycosylphosphatidylinositol-specific phospholipase D1; *IFITM5*, interferon-induced transmembrane protein 5; *LIFR*, leukemia inhibitory factor receptor subunit alpha; *MGP*, matrix Gla protein; *MBTPS2*, membrane-bound transcription factor peptidase, site 2; *NOTCH2*, notch receptor 2; *P3H1*, prolyl 3-hydroxylase 1; *P4HB*, prolyl 4-hydroxylase subunit beta; *PHEX*, phosphate-regulating endopeptidase, X-linked; *PHOSPHO1*, phosphoethanolamine/phosphocholine phosphatase 1; *PLOD2*, procollagen-lysine, 2-oxoglutarate 5-dioxygenase 2; *PLS3*, plastin 3; *PPIB*, peptidylprolyl isomerase B; *PTDSS1*, phosphatidylserine synthase 1; *PTH1R*, parathyroid hormone 1 receptor; *PTH2R*, parathyroid hormone 2 receptor; *RUNX2*, RUNX-family transcription factor 2; *SEC24D*, SEC24 homolog D, COPII coat complex component; *SERPINF1*, serpin family F, member 1; *SERPINH1*, serpin family H, member 1; *SLC34A1*, solute carrier family 34, member 1; *SLC34A3*, solute carrier family 34, member 3; *SLC37A3*, solute carrier family 37, member 3; *SLC39A13*, solute carrier family 39, member 13; *SLC9A3R1*, solute carrier family 9, member 3, regulator 1 (alias *NHERF1*); *SNX10*, sorting nexin 10; *SOX9*, SRY-box transcription factor 9; *SP7*, transcription factor Sp7; *SPARC*, secreted protein, acidic and cysteine-rich; *SPP1*, secreted phosphoprotein 1; *TENT5A*, terminal nucleotidyltransferase 5A; *TMEM38B*, transmembrane protein 38B; *TNFRSF11A*, tumor necrosis family receptor superfamily, member 11a; *TNFRSF11B*, tumor necrosis family receptor superfamily, member 11b; *VDR*, vitamin D receptor; *WNT1*, Wnt family member 1

### Selection of target genes

Several databases, including the Online Mendelian Inheritance in Man (OMIM) database, the Kyoto Encyclopedia of Genes and Genomes (KEGG) Pathway Database, and PubMed, were used to identify a panel of candidate genes that may affect *ALPL* or the HPP phenotype. Specific search terms used to identify genes in peer-reviewed scientific papers and reviews published in the past 30 years included alkaline phosphatase activity, alkaline phosphatase, activity regulation, ALP activity, ALP activity regulation, *ALPL* regulation, HPP disease, and hypophosphatasia. Genes identified in the search of the OMIM database were added to the candidate gene list in the current study if pathogenic variants with perturbation of ALP were known and the mode of inheritance was specified. The KEGG Pathway Database was reviewed to identify upstream and downstream genes involved in ALP pathways; genes identified in this search were added to the list of candidate genes. From these candidates, a joint steering committee of investigators from Alexion, AstraZeneca Rare Disease and Centogene mutually agreed on a final panel of genes for analysis. Genes included in the analysis and their association with diseases similar to HPP are listed in Table 1.

### Data analysis

An end-to-end in-house bioinformatics pipeline, including base calling, primary filtering of low-quality reads and probable artifacts, and annotation of variants was applied. Subsequently, all disease-causing variants reported in the Human Gene Mutation Database [10], ClinVar [11], or CentoMD, as well as variants with minor allele frequency below 1% in the Exome Aggregation Consortium (ExAC) database [12], were considered. In addition to considering all pertinent inheritance patterns, family history and clinical information were used to evaluate identified variants. All identified variants were assessed for their pathogenicity and causality and classified as pathogenic, likely pathogenic, VUS, likely benign, or benign according to the standards and guidelines of the American College of Medical Genetics and Genomics and the Association for Molecular Pathology [13]. All variants related to the patient’s phenotype and CNVs of unknown significance were also reported.

To detect structural variants that might affect *ALPL* but may have been missed with exome sequencing, raw sequence data analysis was performed using the DRAGEN pipeline (Illumina, Inc.); this analysis included base calling, de-multiplexing, alignment to the hg19 human reference genome (Genome Reference Consortium GRCh37), and variant calling. Short-reads were aligned to the GRCh37 (hg19) build of the human reference genome using the DRAGEN aligner algorithm. Variant calling was performed on the alignment files SNVs and indels using Small Variant Caller. Deletions, duplications, inversions, and translocations of at least 50 bp in size were detected using MANTA (Illumina, Inc.) and DRAGEN. Variants were annotated using SnpEff and in-house ad hoc bioinformatics tools.

## Results

### Patients

Of the 16 patients enrolled, 81.3% (*n* = 13) were female. The mean (SD; range) age was 45 (5.4; 36 − 54) years overall, 43 (4.9; 36 − 54) years among female patients, and 51 (1.0; 50 − 52) years among male patients. Table 2 summarizes clinical information reported for each patient.

**Table 2:** Summary of clinical manifestations reported for each patient

Patient Number	Clinical Information
1	Abnormal morphology of thorax, arthralgia, abdominal distension, attention-deficit/hyperactivity disorder, bone pain, decreased bone mineral density, depression, failure to thrive, fatigue, gait disorder, global development delay, headache, joint stiffness, joint swelling, memory impairment, muscle weakness, myalgia, osteopenia, osteoporosis, pain, postural instability, reflux esophagitis, restricted joint mobility, scoliosis, seizure, short stature
2	Arthralgia, abdominal distension, headache, scoliosis
3	Headache, fatigue, muscle weakness, myalgia, postural instability, scoliosis
4	Abnormal thoracic morphology, abdominal distention, arthralgia, bone pain, decreased bone mineral density, fatigue, gait disturbance, headache, joint stiffness, joint swelling, limitation of joint mobility, memory impairment, muscle weakness, myalgia, osteopenia, osteoporosis, pain, pain in joints, postural instability, reflux, reflux esophagitis, scoliosis
5	Abdominal distention, abnormality of teeth, arthralgia, bone pain, chronic pain, delayed ambulatory ability, fatigue, failure to thrive, joint swelling, muscle weakness, myalgia, restriction of joint mobility, scoliosis, seizure
6	Abdominal distention, abnormal alkaline phosphatase level, arthralgia, bone pain, decreased bone mineral density, dyspnea, headache, muscle weakness, pain, postural instability, premature loss of permanent teeth, recurrent fractures
7	Abdominal distention, abnormal alkaline phosphatase level, arthralgia, bone pain, depression, fatigue, gait disturbance, joint stiffness, memory impairment, myalgia, nephrocalcinosis, pain, postural instability, premature loss of teeth, reflux esophagitis, restriction of joint mobility
8	Abdominal distention, arthralgia, body aches, bone pain, fatigue, headache, memory impairment, muscle weakness, myalgia, postural instability
9	Abdominal distention, arthralgia, attention-deficit/hyperactivity disorder, bone pain, fatigue, memory impairment, muscle weakness, myalgia, reflux esophagitis, seizure, scoliosis
10	Abdominal distention, abnormality of calvarial morphology, arthralgia, bone pain, chronic pain, decreased bone mineral density, fatigue, joint swelling, premature loss of deciduous teeth, recurrent fractures, reflux esophagitis
11	Abdominal distention, arthralgia, body aches, depression, joint stiffness, fatigue, muscle weakness, myalgia, premature loss of permanent teeth, reflux esophagitis, restriction of joint mobility, scoliosis
12	Abdominal distention, abnormal thoracic morphology, arthralgia, attention-deficit/hyperactivity disorder, bone pain, chronic pain, decreased bone mineral density, fatigue, headache, joint swelling, kidney stone, limitation of joint mobility, muscle weakness, pain, reflux esophagitis, rickets, scoliosis
13	Abdominal distention, arthralgia, attention-deficit/hyperactivity disorder, bone pain, decreased body weight, depression, dysphagia, fatigue, gait disturbance, headache, joint stiffness, loss of appetite, memory impairment, muscle weakness, myalgia, restricted joint mobility, scoliosis, seizure
14	Arthralgia, body aches, bone pain, decreased bone mineral density, fatigue, joint stiffness, kidney stone, nephrocalcinosis, osteopenia, osteoporosis, pain, recurrent fractures, restriction of joint mobility, seizure
15	Arthralgia, attention-deficit/hyperactivity disorder, body aches, bone pain, depression, fatigue, joint stiffness, memory impairment, muscle weakness, myalgia, pain, postural instability, restricted joint mobility, scoliosis
16	Abdominal distention, arthralgia, bone pain, body aches, decreased bone mineral density, depression, failure to thrive, fatigue, joint stiffness, muscle weakness, myalgia, osteopenia, osteoporosis, pain, restricted joint mobility

### Clinical chemistry

**Fig. 1:**
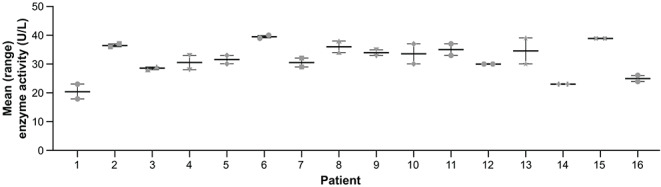
ALP enzyme activity.^a a^Normal range: females 42–98 U/L, males 53–128 U/L. ALP, alkaline phosphatase

Figure 1 shows mean (range) serum ALP activity for each patient measured on 2 occasions at least 1 month apart. Mean (SD; range) ALP enzyme activity was 30.81 (5.68; 17.90 − 40.0) U/L for the first measurement and 32.62 (5.80; 23.0 − 39.0) U/L for the second measurement. All measurements were below the normal age- and sex-adjusted range for ALP enzyme activity for adult males (53–128 U/L) and females (42–98 U/L) [14].

### Genetic testing

All 16 patients received a genetic report and were confirmed by WGS to be negative for clinically relevant *ALPL* variants. Table 1 lists genes that were identified through OMIM search as regulators of *ALPL* or the HPP phenotype. Four patients (25%; patients 3, 4, 7, and 15) had positive WGS findings for variants in other genes that may interfere with ALP enzyme activity or result in some clinical manifestations mimicking HPP (Table 3).

**Table 3:** Summary of positive results for genetic variants in genes whose products may regulate ALP activity or modify HPP phenotype

Pt no.	Sex	Age, y	Patient Symptoms	Gene and Function	Positional Variant and Zygosity	Classification	Interpretation	Clinical Symptoms Associated With Identified Gene Variant
3	F	54	Headache, fatigue, muscle weakness, myalgia, postural instability, scoliosis	*COL1A1* Pro-alpha1 chains of collagen triple helix	c.642 + 16 A > G Heterozygous	VUS	Osteogenesis imperfecta types 1–4, Ehlers-Danlos syndrome type VIIA and classical subtype, Caffey disease, idiopathic osteoporosis	Low bone density, bone fragility, long bone fractures, fractures with minimal trauma, joint and skin hyperextensibility, soft tissue swelling over affected bones
				*NLRP12* Attenuating factor of inflammation	c.727 A > G (p.Arg243Gly) Heterozygous	VUS	Familial cold autoinflammatory syndrome type 2	Episodic and recurrent rash, urticaria, arthralgia, myalgia, headache
				*SCN9A* Voltage-gated sodium channel involved in nociception signaling	Deletion of chr2:167157718–167462541 Heterozygous	VUS	Primary erythromelalgia, paroxysmal extreme pain disorder, channelopathy-associated insensitivity to pain	Recurrent episodes of intense, burning pain and redness, warmth, swelling; insensitivity to pain
4	F	43	Abnormal thoracic morphology, arthralgia, abdominal distention, gait disturbance, memory impairment, joint stiffness, joint swelling, bone pain, headache, limitation of joint mobility, fatigue, muscle weakness, myalgia, osteopenia, osteoporosis, postural instability, decreased bone mineral density, reflux, pain in joints, reflux esophagitis, pain, scoliosis	*P3H1* Member of the collagen prolyl hydroxylase family	c.1346-1G > A Heterozygous	Likely pathogenic	Osteogenesis imperfecta type 8 autosomal recessive inheritance	Connective tissue disorder, increased bone fragility, low bone mass, tendency toward fractures
7	F	36	Abnormal ALP activity, arthralgia, abdominal distention, depression, gait disturbance, memory impairment, joint stiffness, bone pain, restriction of joint mobility, fatigue, myalgia, nephrocalcinosis, postural instability, premature loss of teeth, reflux esophagitis, pain	*SGCE* Transmembrane protein that links the actin skeleton to the extracellular matrix	c.817 C > T Premature stop codon Heterozygous	Pathogenic; *SGCE* locus is known to be imprinted	Myoclonus dystonia type 11 autosomal dominant inheritance	Rare movement disorder with mild to moderate dystonia
15	M	51	Arthralgia, attention-deficit/hyperactivity disorder, body aches, depression, memory impairment, joint stiffness, bone pain, restricted joint mobility, fatigue, muscle weakness, myalgia, postural instability, pain, scoliosis	*VDR* Vitamin D receptor superfamily	c.265 A > G (p.Met89Val) Heterozygous	VUS	Vitamin D–dependent rickets type 2 A, autosomal recessive inheritance	Characterized by hypocalcemia, severe rickets, and alopecia

ALP, alkaline phosphatase; HPP, hypophosphatasia; VUS, variant of uncertain significance

Genes: *COL1A1*, collagen, type I, alpha-1 chain; *NLRP12*, nod-like receptor pyrin domain containing 12; *P3H1*, prolyl 3-hydroxylase 1; *SCN9A*, sodium voltage-gated channel alpha subunit 9; *SGCE*, sarcoglycan epsilon; *VDR*, vitamin D receptor

Patient 3 (54-year-old woman) was found to have a heterozygous intronic VUS in the splice region of the *COL1A1* gene on chromosome 17 (genomic location, 48275294T > C; variant, NM_000088.3:c.642 + 16 A > G), which codes for collagen, type I, alpha-1 protein. Additionally, testing outside the requested gene panel showed a heterozygous missense VUS in *NLRP12* on chromosome 19 (genomic location, 54314186T > C; variant, NM_001277126.1:c.727 A > G NM_001277126.1 [p.Arg243Gly]), coding for the nucleotide-binding leucine-rich repeat-containing receptor 12, as well as a CNV (heterozygous deletion) of *SCN9A*, coding for sodium voltage-gated channel alpha subunit 9, from chromosome 2 (genomic location, 167157718–167362541).

Patient 4 (43-year-old woman) was found to be a heterozygous carrier of a likely pathogenic variant (NM_001243246.1:c.1346-1G > A) in the *P3H1* gene, which codes for prolyl 3-hydroxylase 1, a member of the collagen prolyl hydroxylase family implicated in autosomal recessive osteogenesis imperfecta [15]. Testing outside the requested gene panel showed that Patient 7 (36-year-old woman) had a heterozygous pathogenic variant (NM_001346713.1:c.817 C > T, leading to a premature stop codon) in *SGCE*, coding for sarcoglycan epsilon, a transmembrane protein that links the cytoskeleton protein, actin, to the extracellular matrix. This *SGCE* variant is involved in myoclonus dystonia syndrome (MDS) type 11, a disease with autosomal dominant inheritance that results in rapid, involuntary muscle jerks. No other variant relevant to the described phenotype was found. Patient 15 (51-year-old man) had a heterozygous VUS (positional mutation, NM_001017536.1:c.265 A > G, [p.Met89Val]) in *VDR*, which codes for the vitamin D receptor.

## Discussion

HPP is a disabling condition with wide-ranging impacts on health-related quality of life [16]. However, accurate diagnosis and appropriate treatment are often delayed, owing, in part, to symptom overlap between HPP and more common conditions [1, 17]. Sequencing of the *ALPL* coding region confirms the diagnosis of HPP if an *ALPL* variant is identified; however, some patients with clinical and laboratory findings consistent with a diagnosis of HPP have negative genetic testing results [4].

The current study was performed to further elucidate the genetic underpinnings of *ALPL* and non-*ALPL* variants beyond what can be detected with standard sequencing of the *ALPL* coding region, including genes that may interfere with *ALPL* expression or processing, or activity of ALP, thus modifying the phenotypic presentation of HPP.

With regard to the primary objective of this study, WGS did not reveal any additional variants in *ALPL* that, according to the current knowledge, were considered pathogenetically relevant and caused reduced ALP activity and HPP symptoms in these patients. However, low ALP activity may be attributed to variation in the non-coding region of *ALPL*, since there is no definition of what constitutes normal *ALPL* intronic sequence vs. pathogenic variations in *ALPL* intronic sequences.

Results of this study with respect to the secondary objective of identifying novel genetic variants beyond *ALPL* that could be pathogenetically relevant do not provide an answer to the long-standing speculation that confounding genes modulate impact on either *ALPL* expression or ALP activity. Although we have observed variations in several genes that were a priori selected as potentially responsible for low ALP activity, or demonstrated some phenotypic overlap with HPP, we do not claim these to be causative of HPP. The mechanisms involved in this observed association remain unknown. Further research is needed to better understand the molecular basis of HPP in these *ALPL* variant-negative patients.

All 16 patients with clinical and laboratory results indicative of HPP were confirmed by WGS to be negative for pathogenic *ALPL* variants, including rare, non-coding variants with potential implications in gene splicing and gross copy number alterations. However, 4 of the 16 patients had findings for genetic variants in other genes with the potential for regulating ALP activity or modifying the HPP phenotype. Variants in *COL1A1* are associated with bone disorders, including osteogenesis imperfecta types 1–4 [18]. Osteogenesis imperfecta is a connective tissue disorder associated with bone fragility, making it a potential differential diagnosis of HPP [1, 19]. Variants in *SCN9A* are associated with primary erythromelalgia, paroxysmal extreme pain disorder, or insensitivity to pain [20], and variants in *NLRP12* are associated with familial cold-induced autoinflammatory syndromes [21]. Patient 3 reported history of pain and headache, which could correspond with these conditions but are nonspecific. Since this patient had no history of other, more specific manifestations or clinical presentations associated with these disorders, such as low bone mineral density, rash, redness, warmth, or swelling, the patient was not diagnosed with any of these disorders.

The *P3H1* variant NM_001243246.1:c.1346-1G > A is a splice variant known to impair the function of the nearby acceptor splice site, and a variant at this position (with a different nucleotide exchange, c.1346-1G > C, ClinVar variation ID: 284532, clinical testing, listed as pathogenic) has been reported as disease-causing for autosomal recessive osteogenesis imperfecta type 8 [15]. However, the patient’s clinical phenotype was not consistent with osteogenesis imperfecta type 8 and a second relevant variant in *P3H1* compatible with an autosomal-recessive trait was not demonstrable. Therefore, the patient is currently deemed to be an asymptomatic carrier of a pathogenic *P3H1* variant.

The pathogenic variant in the *SGCE* gene (NM_001346713.1:c.817 C > T) leads to a premature stop codon and has been described as pathogenic for MDS (OMIM: 159900), a rare movement disorder characterized by dystonia and lightning-like myoclonic jerks [22]. As the *SGCE* locus is imprinted, approximately 95% of patients with MDS who inherit the variant from their mother will remain healthy, whereas almost all children who inherit the variant from their father will develop MDS [22]. De novo variants are also described. Since the clinical picture of MDS has little overlap with that of the patient and cannot explain her symptoms (e.g., premature loss of permanent teeth), this variant was likely inherited from the mother and thus does not result in the typical phenotype of MDS.

*VDR* encodes the vitamin D receptor, a member of the nuclear hormone receptor superfamily of ligand-inducible transcription factors [23]. The identified variant NM_001017536.1:c.265 A > G (p.Met89Val) leads to an amino acid exchange from Met to Val at position 89. Pathogenic variants in *VDR* are associated with vitamin D–resistant rickets type 2A, a disease with an autosomal recessive inheritance [24]. Vitamin D–resistant rickets type 2A or hypocalcemic vitamin D–resistant rickets is an inherited disorder of vitamin D characterized by hypocalcemia, severe rickets, and, in many cases, alopecia [24]. Although not directly related to *ALPL*, variants in *VDR* may interact with *ALPL* at some level and influence the phenotypic presentation of the disease.

The current study is not without limitations. The small study population was overrepresented by females from a single geographic region, reducing generalizability of the results. Accordingly, results of this analysis highlight the importance of future genomic studies in larger cohorts from a variety of geographic regions, including additional genes that may contribute to HPP manifestations, to better understand the genetic basis of HPP. In addition, future studies should investigate epigenetic and extragenomic factors via transcriptomic and metabolomics analyses.

## Conclusions

The genetic basis of HPP in patients without *ALPL* variants remains unclear. In the present analysis, all patients were confirmed via WGS to be negative for variants in *ALPL* despite low ALP activity. Variants in non-*ALPL* genes did not conclusively overlap with genes associated with any other disorder and may contribute to the HPP phenotype through unknown mechanisms. These data provide an important rationale for further investigation of molecular mechanisms linking genes beyond *ALPL* to the HPP phenotype and of the genetic underpinnings of HPP. Nevertheless, diagnosis of HPP remains a clinical diagnosis based on signs and symptoms in affected individuals and does not require detection of *ALPL* variants [1].

## Data Availability

Alexion, AstraZeneca Rare Disease will consider requests for disclosure of clinical study patient-level data provided that patient privacy is assured through methods like data de-identification, pseudonymization, or anonymization (as required by applicable law), and if such disclosure was included in the relevant study informed consent form or similar documentation. Qualified academic investigators may request patient-level clinical data and supporting documents (statistical analysis plan and protocol) pertaining to Alexion-sponsored studies. Further details regarding data availability and instructions for requesting information are available in the Alexion Clinical Trials Disclosure and Transparency Policy at https://alexion.com/our-research/research-and-development. Link to Data Request Form (https://alexion.com/contact-alexion/medical-information).

## Abbreviations

ALP: Alkaline Phosphatase
CNV: Copy Number Variants
ExAC: Exome Aggregation Consortium
GWAS: Genome-Wide Association Study
HPP: Hypophosphatasia
KEGG: Kyoto Encyclopedia of Genes and Genomes
MDS: Myoclonus Dystonia Syndrome
OMIM: Online Mendelian Inheritance in Man
PLP: Pyridoxal 5ʹ-Phosphate
PPi: inorganic Pyrophosphate
SD: Standard Deviation
SNV: Single Nucleotide Variants
VUS: Variants of Uncertain Significance
WGS: Whole Genome Sequencing
